# Supplementary material for: Multi-Omics Characterization of Early- and Adult-Onset Major Depressive Disorder
Source: J Pers Med. 2022 Mar 6;12(3):412. doi: 10.3390/jpm12030412 (PMC8949112; doi:10.3390/jpm12030412)
Supplement: Supplementary file 1 [file jpm-12-00412-s001.zip › 22_03_08_supplementary.pdf]

Supplementary Materials

TITLE: Multi-Omics Characterization of Early and Adult-Onset Major Depressive Disorder

PREVIOUS PRESENTATION: None

AUTHORS: Caroline W. Grant[1], Erin F. Barreto[2], Rakesh Kumar[3], Rima Kaddurah-Daouk[4], Michelle Skime[3], Taryn Mayes[5], Thomas Carmody[6], Joanna Biernacka[7], Liewei Wang[1], Richard Weinshilboum[1], Madhukar H. Trivedi[5], William V. Bobo[8], Paul E. Croarkin[3#], Arjun P. Athreya[1#]

AUTHOR AFFILIATIONS
1. Department of Molecular Pharmacology and Experimental Therapeutics, Mayo Clinic, Rochester, MN, USA.
2. Department of Pharmacy, Mayo Clinic, Rochester, MN, USA.
3. Department of Psychiatry and Psychology, Mayo Clinic, Rochester, MN, USA.
4. Department of Psychiatry and Behavioral Sciences, Department of Medicine, Duke Institute for Brain Sciences, Duke University, Durham, NC, USA.
5. Peter O'Donnell Jr. Brain Institute and the Department of Psychiatry at the University of Texas Southwestern Medical Center, Dallas, TX, USA.
6. Department Population and Data Sciences at the University of Texas Southwestern Medical Center in Dallas, Dallas, TX, USA.
7. Department of Quantitative Health Sciences, Mayo Clinic, Rochester, MN, USA.
8. Department of Psychiatry and Psychology, Mayo Clinic, Jacksonville, FL, USA.

# These authors are co-corresponding authors.

Corresponding author:
Arjun P. Athreya, M.S., PhD.,
Dept. of Molecular Pharmacology and Exp. Therapeutics, Mayo Clinic
200 First St. SW,
Rochester, MN – 55902
Tel: +1-507-422-6073

KEYWORDS: Genomics, metabolomics, major depressive disorder, age at onset

FIGURES

Figure Legends

Supplementary Figure S1. CO-MED sample inclusion for GWAS and Multi-Omics Integration Analysis

Supplementary Figure S2. PGRN-AMPS sample inclusion for genomic replication and Multi-Omics Integration Analysis

Supplementary Figure S3. GWAS QQ-Plot for A) PGRN-AMPS and B) CO-MED with assessment of genomic inflation. GC Lambda 0.5: Genomic Control lambda calculated based on the 50th percentile (median).

Supplementary Figure S4. GWAS locus zoom plots for variants retained following multi-omics integration analysis.

TABLES

Table Legends

Supplementary Table S1. Metabolites Common to PGRN-AMPS and CO-MED and Included in Multi-Omics Integration Analysis.

Supplementary Table S2. PGRN-AMPS cohort GWAS top SNVs. GWAS associations with age of depressive onset ($p < 1E-5$). Nearest genes, distance from nearest genes, and locations are based upon the NCBI Reference Sequences (RefSeq) database, as queried using haploR. Distances are reported in base-pair units. eQTL genes and tissues were annotated using haploR and the Genotype-Tissue Expression (GTEx) version 6 for cis eQTLs, and results of Westra et al., (2013) for trans eQTLs. Locations denoted by '.' indicate that the SNV is outside the open reading frame. Negative betas indicate association of the minor allele with earlier age of onset, while positive betas indicate association of the minor allele with later age of onset.

Supplementary Table S3. CO-MED cohort GWAS top SNVs. A. GWAS associations with age of depressive onset ($p < 1E-5$). B. Sensitivity analysis for CO-MED cohort GWAS excluding one individual with self-reported age at depressive onset of 0. Nearest genes, distance from nearest genes, and locations are based upon the NCBI Reference Sequences (RefSeq) database, as queried using haploR. Distances are reported in base-pair units. eQTL genes and tissues were annotated using haploR and the Genotype-Tissue Expression (GTEx) version 6 for cis eQTLs, and results of Westra et al., (2013) for trans eQTLs. Locations denoted by '.' indicate that the SNV is outside the open reading frame. Negative betas indicate association of the minor allele with earlier age of onset, while positive betas indicate association of the minor allele with later age of onset.

Supplementary Table S4. Functional Annotation via DAVID.

Supplementary Table S5: Multi-Omics Integration Network Correlations and P-Values. Pearson correlation coefficient and p-value for significant associations ($|r| > 0.1$, $p < 0.05$) between SNVs and metabolites represented in either or both networks (pre-adult and adult onset MDD). For descriptive purposes, SNVs are labelled by rsID and genomic annotations for the nearest gene annotated by the NCBI RefSeq Database.

Supplementary Figure S1: CO-MED sample inclusion for GWAS and Multi-Omics Integration Analysis

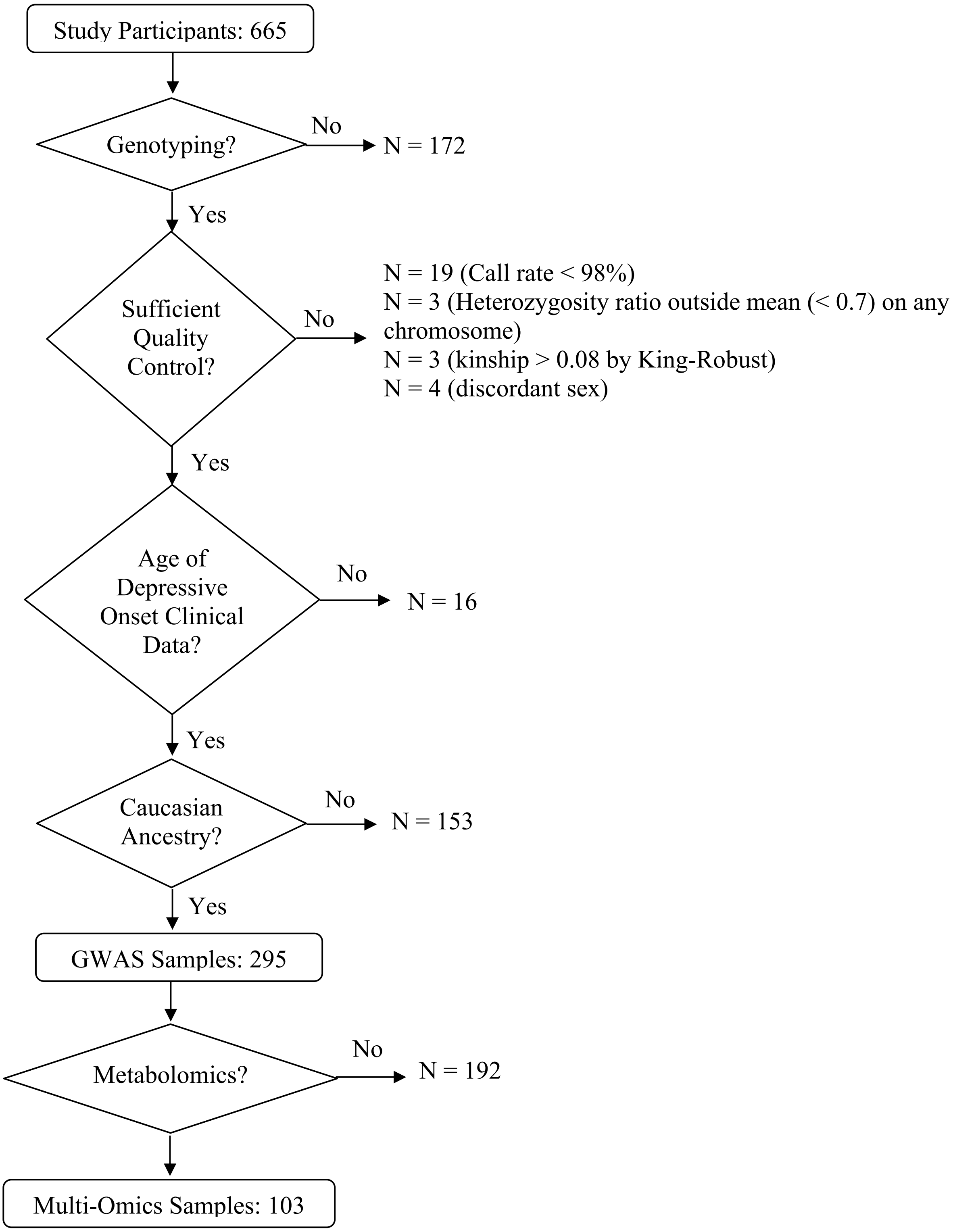

Supplementary Figure S2: PGRN-AMPS sample inclusion for genomic replication and Multi-Omics Integration Analysis

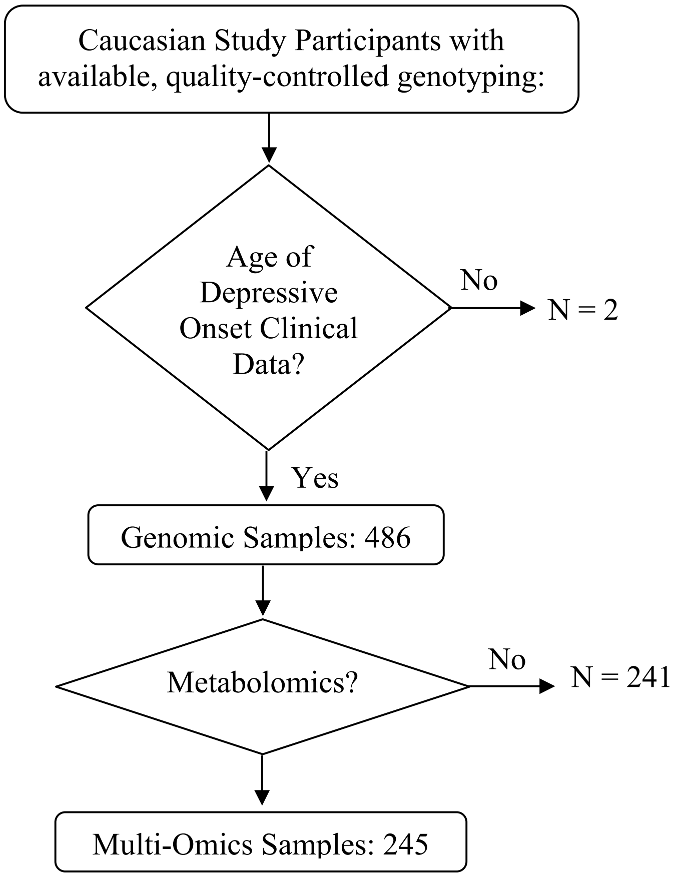

Supplementary Figure S3: GWAS QQ-Plot.

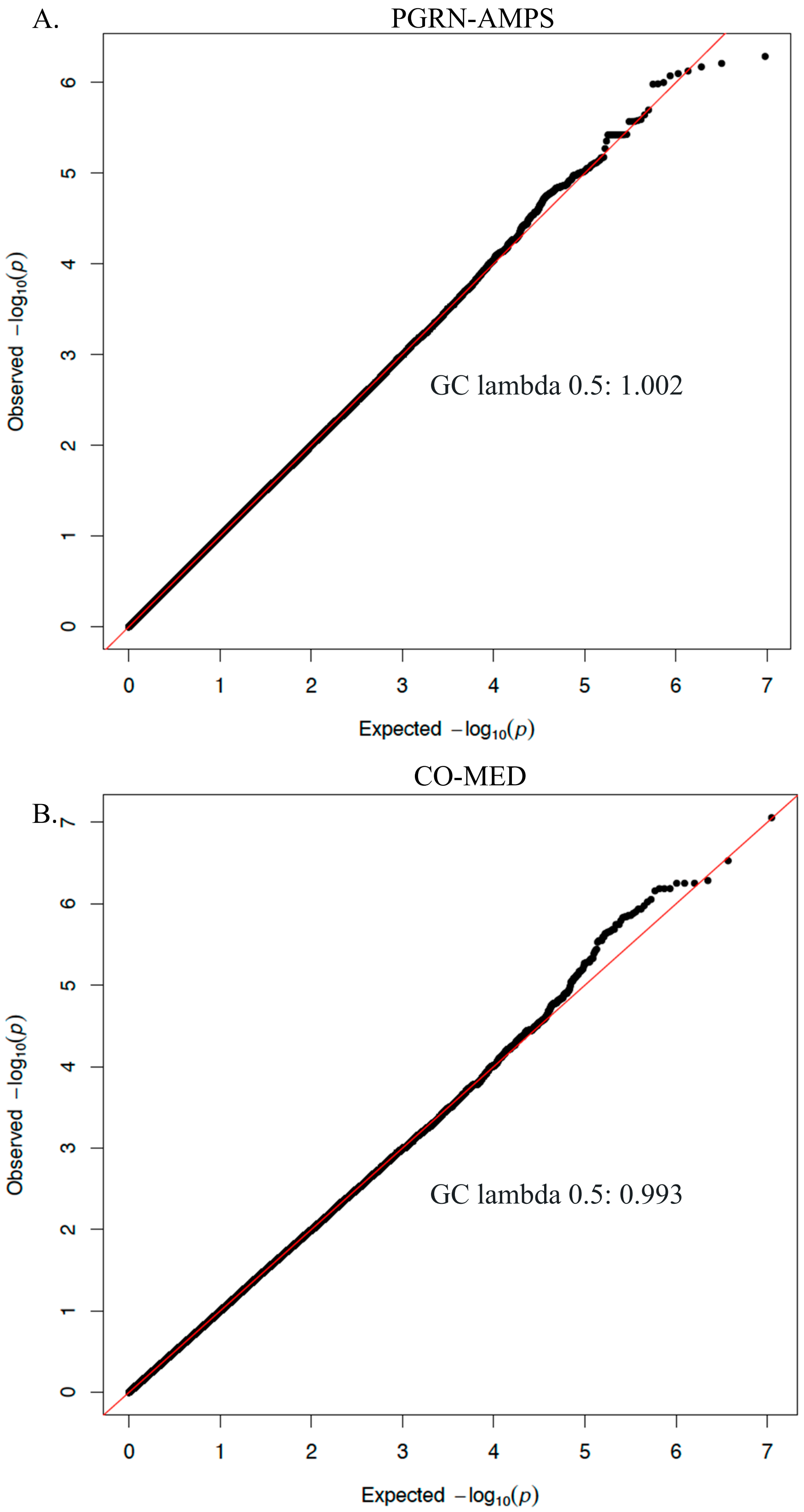

Supplementary Figure S4: GWAS locus zoom plots for variants retained following multi-omics integration analysis.

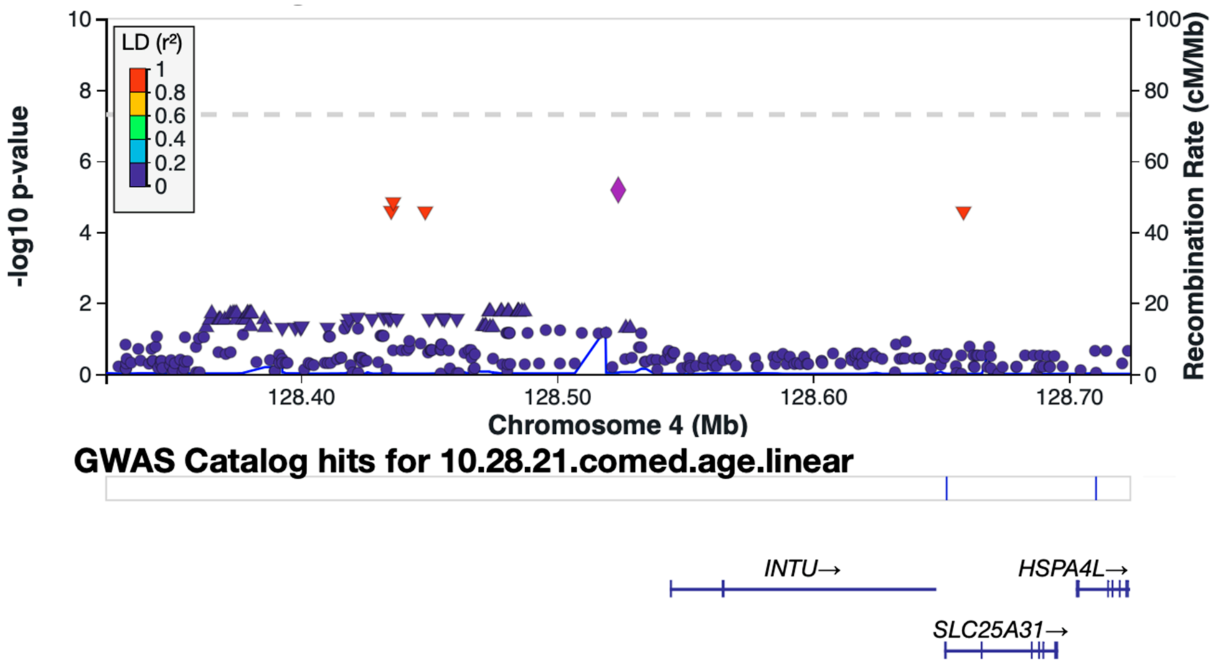

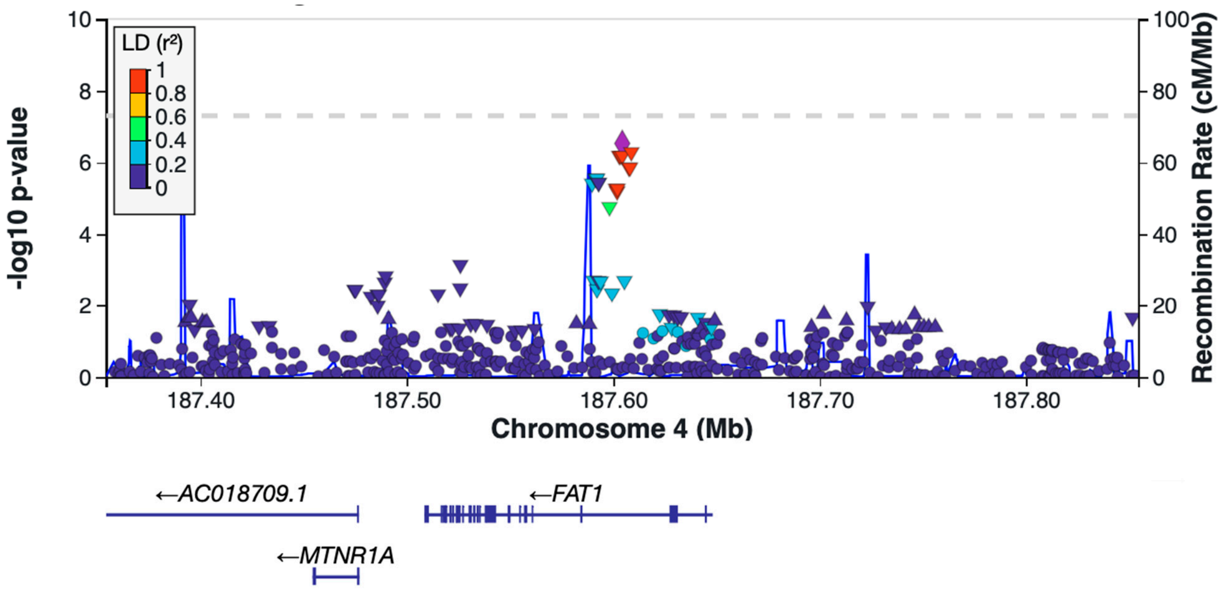

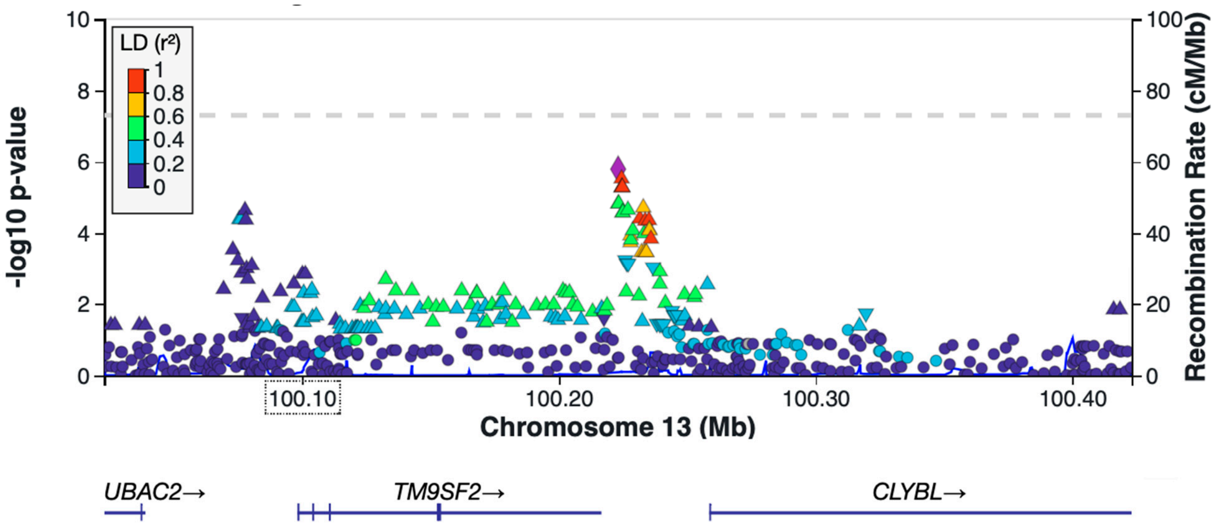
LD (r²)
1
0.8
0.6
0.4
0.2
0
-log10 p-value
Recombination Rate (cM/Mb)
100.10
100.20
100.30
100.40
Chromosome 13 (Mb)
UBAC2→
TM9SF2→
CLYBL→

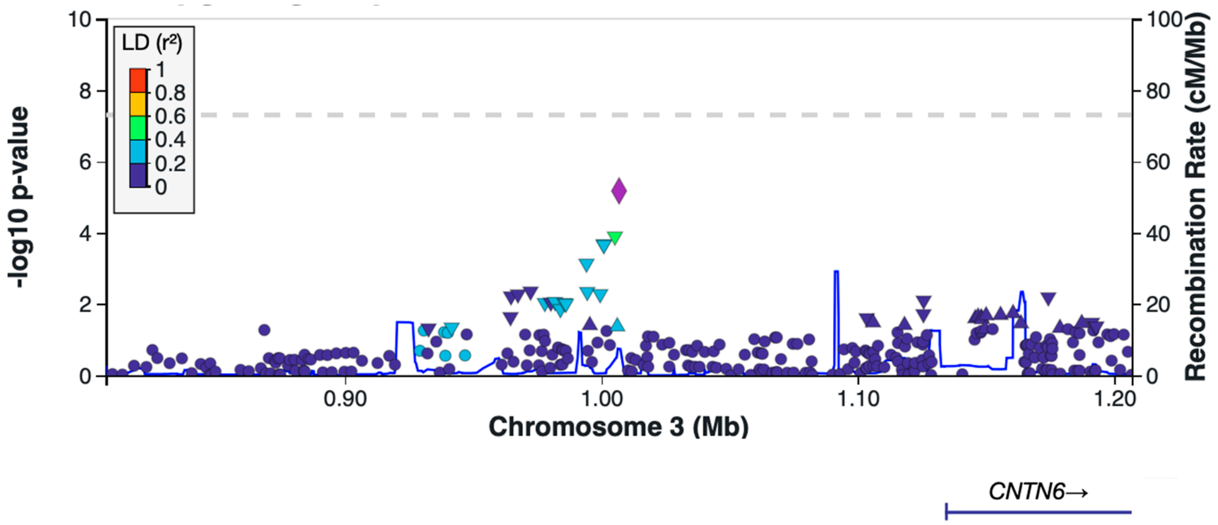
LD (r²)
1
0.8
0.6
0.4
0.2
0
-log10 p-value
Recombination Rate (cM/Mb)
0.90
1.00
1.10
1.20
Chromosome 3 (Mb)
CNTN6→

Supplementary References

Westra, H. J., M. J. Peters, T. Esko, H. Yaghootkar, C. Schurmann, J. Kettunen, M. W. Christiansen*, et al.* "Systematic Identification of Trans Eqtls as Putative Drivers of Known Disease Associations." *Nat Genet* 45, no. 10 (Oct 2013): 1238-43. https://doi.org/10.1038/ng.2756. https://www.ncbi.nlm.nih.gov/pubmed/24013639.
